# Modification of Neural Circuit Functions by Microglial P2Y6 Receptors in Health and Neurodegeneration

**DOI:** 10.1007/s12035-024-04531-8

**Published:** 2024-10-14

**Authors:** Yi Zhang, Yong Tang, Peter Illes

**Affiliations:** 1 International Joint Research Centre on Purinergic Signaling, School of Acupuncture and Tuina, Chengdu University of Traditional Chinese Medicine, Chengdu, China; 2 Acupuncture and Chronobiology Key Laboratory of Sichuan Province, Chengdu, China; 3 Rudolf Boehm Institute for Pharmacology and Toxicology, University of Leipzig, Leipzig, Germany; 4 School of Health and Rehabilitation, Chengdu University of Traditional Chinese Medicine, Chengdu, China

**Keywords:** Neural circuits, P2Y6 receptors, Microglial phagocytosis, Synaptic pruning, Transmitter release and uptake, Neurodegenerative diseases

## Abstract

Neural circuits consisting of neurons and glial cells help to establish all functions of the CNS. Microglia, the resident immunocytes of the CNS, are endowed with UDP-sensitive P2Y6 receptors (P2Y6Rs) which regulate phagocytosis/pruning of excessive synapses during individual development and refine synapses in an activity-dependent manner during adulthood. In addition, this type of receptor plays a decisive role in primary (Alzheimer’s disease, Parkinson’s disease, neuropathic pain) and secondary (epilepsy, ischemic-, mechanical-, or irradiation-induced) neurodegeneration. A whole range of microglial cytokines controlled by P2Y6Rs, such as the interleukins IL-1β, IL-6, IL-8, and tumor necrosis factor-α (TNF-α), leads to neuroinflammation, resulting in neurodegeneration. Hence, small molecular antagonists of P2Y6Rs and genetic knockdown of this receptor provide feasible ways to alleviate inflammation-induced neurological disorders but might also interfere with the regulation of the synaptic circuitry. The present review aims at investigating this dual role of P2Y6Rs in microglia, both in shaping neural circuits by targeted phagocytosis and promoting neurodegenerative illnesses by fostering neuroinflammation through multiple transduction mechanisms.

## Introduction

The neural circuit is a complex network of neurons and glial cells that facilitates the transmission and integration of neural information, underpinning the fundamental operation of the nervous system [1–3]. The main populations of glia consist of neuroglia (astrocytes and oligodendrocytes) as well as microglia [4, 5]. As resident immune cells of the CNS, microglia not only maintain the stability of the intracerebral environment, but also play a key role in the establishment, maintenance, and remodeling of neural circuits [6–8].

Purinergic signaling by ATP/ADP, UTP/UDP, and adenosine serves as a crucial mechanism governing the dynamic interplay between neurons and glial cells [9, 10] with the P2Y6 receptor (P2Y6R) emerging as one of the most prominently expressed purinergic receptor on microglia [11]. This receptor primarily responds to its endogenous ligand, uridine diphosphate (UDP), and orchestrates the biological functions of microglial cells by initiating G-protein signaling pathways. Notably, the P2Y6R plays a key role in regulating microglial cell activation, migration, and the first-line phagocytic capabilities [12]. Moreover, P2Y6R plays a role in the turnover of neurotransmitters [13] Activation of P2Y6Rs has been demonstrated to facilitate optimal brain structure and function. Conversely, dysregulation of P2Y6R signaling has been shown to disrupt the delicate balance between synaptic formation and clearance, which may in turn contribute to the etiology of developmental disorders or neurodegenerative diseases.

Despite the growing interest in microglial P2Y6Rs as a potential target for CNS disorders, their role in regulating neural circuits remains understudied [14]. The aim of the present review was twofold: Firstly, we have set out to review the crucial functions of microglia in shaping, via P2Y6R stimulation, neural circuits during development and adulthood. Secondly, we intended to decipher the role of P2Y6Rs as pathological factors in neurodegenerative diseases.

## P2Y6 Receptor

The P2Y6R, a G protein-coupled receptor, predominantly expressed on microglial cells both in the central and peripheral nervous systems, responds to its endogenous ligand UDP [12]. UTP is released from neurons through pannexin channels or connexin hemichannels in response to stress or injury and can be enzymatically converted to UDP by ectonucleotidases [15, 16]. UDP acts as a “find me” signal, that activates P2Y6Rs, causing microglia to migrate to damaged neurons and upregulate the formation of their large vacuoles, thereby enhancing phagocytosis [17]. Upon activation of the P2Y6R, downstream signaling pathways involving G proteins are engaged, culminating in the activation of phospholipase C (PLC), protein kinase C (PKC), and the subsequent regulation of cytoskeletal reorganization and phagosome formation [18, 19]. Noteworthy research has highlighted that P2Y6R activation can elevate intracellular calcium levels, thereby activating calcium-dependent signaling cascades, such as calcium/calmodulin-dependent protein kinase (CAMK), to enhance phagocytosis [20, 21]. Purinergic signaling regulates calcium wave–mediated synaptic events, as calcium ions can excite multiple signaling pathways within neurons, affecting synaptic strength and transmission efficiency between presynaptic and postsynaptic neurons [22].

Activation of the P2Y6R may also modulate the expression of proinflammatory cytokines by regulating nuclear factor kappa-light-chain-enhancer of activated B cells (NF-κB), leading to increased secretion of inflammatory mediators like interleukin 1β (IL-1β) and tumor necrosis factor-α (TNF-α) from microglial cells. This enhanced inflammatory response can further potentiate phagocytic activity, potentially resulting in neuronal loss under inflammatory conditions [23–26]. Inflammation-mediated synaptic phagocytosis has the capacity to either fortify or weaken neuronal synaptic strength, thereby impacting information transmission between neurons [27]. Studies have shown that P2Y6R activation significantly upregulates lysosomes in key brain regions such as the hippocampus, amygdala, and thalamus. Genetic knockout of P2Y6Rs in mice leads to reduced lysosomal expression in these regions, suggesting that P2Y6R activation may facilitate lysosomal fusion and augment the degradation and phagocytic capabilities of microglial cells [28, 29].

## Microglial P2Y6 Receptors and Neural Circuits

Neural circuits serve as the fundamental framework for all brain activities and intricate behaviors [30]. Throughout ontogenesis, neurons establish precise connections to culminate in mature central nervous system circuits. Microglia play indispensable roles in synaptic formation, modification, maturation, and also neurogenesis [31]. P2Y6Rs regulate neural circuit plasticity by inducing synaptic phagocytosis and neurotransmitter uptake, thus emphasizing the critical role of P2Y6Rs in regulating microglial function within neural circuits.

### Involvement of Microglia in the Plasticity Processes of Neural Circuits

As sentinels of the CNS, microglia not only monitor and regulate immune responses, but also play important roles in the secretion of cytokines, the release of neurotrophic factors, the phagocytosis of neurons and cell debris, and the formation and pruning of synapses [32–34]. Microglia form close connections with neurons and other glial cells, working together to weave complex cellular networks [35, 36]. Microglia refine synapses in an activity-dependent manner; this is an ongoing adaptive process that responds to the dynamic brain microenvironment and ensures homeostasis and balance within neural circuits [37]. During the formation of neural circuits, microglia modulate the state of neuronal activity, by providing essential nutrients and growth factors critical for the functioning of neurons.

In the resting state, microglia maintain surveillance and form contacts with neurons through dynamic extension and retraction of their processes [32]. This is regulated by another type of G protein–coupled receptor, the P2Y12R, which is located at the microglial processes and senses the damage [38, 39] or activity-dependent release of ATP/ADP from nerve terminals [40, 41]. Microglia secretes brain-derived neurotrophic factor (BDNF), which can increase the phosphorylation of tropomyosin-related kinase receptor B (TrkB, a key mediator of synaptic plasticity, which is considered to represent a cellular model of learning and memory), thereby affecting cognitive functions [42, 43]. In addition, microglia act as scavengers of neural circuit constituents, participating in the dynamic process of synapse formation and selective elimination [44].

Microglia are involved in the precise regulation of synaptic pruning throughout neuronal development by recruiting specific instructive or permissive signals, such as the complement receptor 3 (CR3)/C3 pathway and calcium-dependent purinergic signaling pathways [35]. Studies have shown that a type 1 interferon (IFN-1)-responsive microglial state that actively engulfs entire neurons has been identified in the developing somatosensory cortex on postnatal day 5. These data identify an important role of microglia engulfing neurons during a critical time window of brain development [45]. This selective elimination of redundant synapses [34] in developing neural circuits promotes synaptic remodeling and neuronal maturation, thereby influencing the configuration of neural networks to optimize the establishment and development of neural connections in the brain [46].

### P2Y6 Receptor Regulates Synaptic Pruning

The developing nervous system undergoes significant remodeling to achieve the high precision of mature neural circuits. This precision is achieved through pruning — an extensive process in which axons, dendrites, and synapses that initially were formed in excessive numbers become eliminated [47, 48]. Synaptic pruning occurs not only during development but also throughout adulthood and refers to the removal of certain synaptic components, including presynaptic terminals/postsynaptic membranes, and may extend to the elimination of axonal and dendritic debris [49]. At the same time, this process involves the preservation, strengthening, and maturation of remaining synapses [50]. Microglia, the brain’s specialized phagocytic cells, are responsible for the clearance of apoptotic cells and actively promote cellular regeneration throughout development, aging, and neurodegenerative diseases [51]. Microglia are key to identifying and eliminating redundant neural connections, thereby promoting the refinement of neural circuits and optimizing the efficiency of neuronal networks [52, 53].

**Fig. 1:**
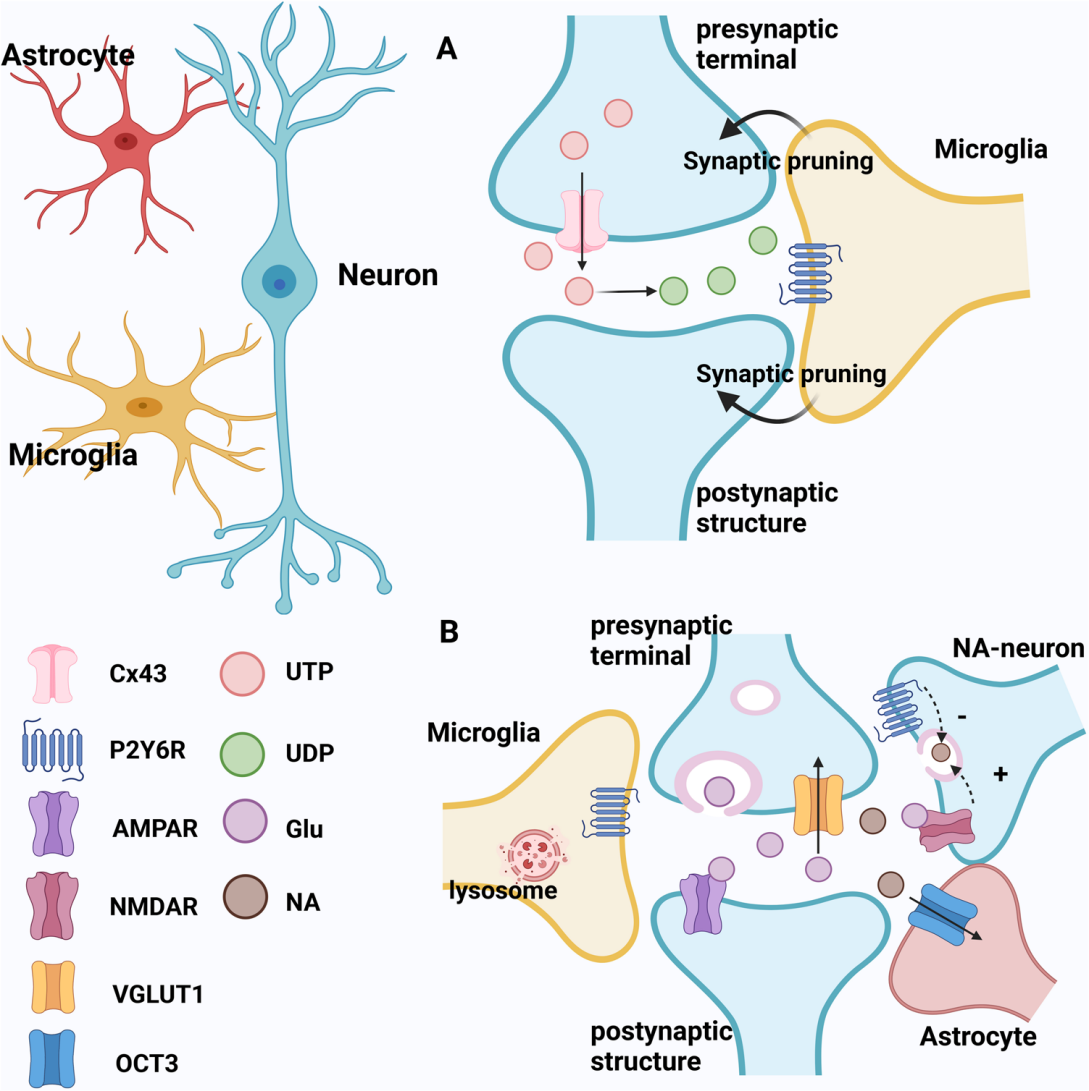
The proposed mechanisms of P2Y6Rs to regulate neuronal circuit plasticity is by inducing synaptic phagocytosis by microglia and modulation of neurotransmitter release/uptake. (**A**) Neurons release UTP through connexin hemichannels (Cx43); this UTP can be converted by ectonucleotidases to UDP, which acts as a 'find me' signal via microglial P2Y6Rs, mediating pruning of unwanted synapses by microglia and thereby regulating neuronal connectivity. At the same time UTP can also originate from any type of damaged CNS cell (neuronal or non-neuronal). (**B**) Glutamate released from glutamatergic neurons activates the presynaptic NMDARs of noradrenergic neurons, leading to the release of this transmitter. P2Y6Rs located at the noradrenergic nerve terminals inhibit the release of noradrenaline (NA). Microglial P2Y6Rs indirectly regulate the release of NA by enhancing its uptake into astrocytes (uptake 2; organic cationic transporter 3; OCT3), also decreasing the synaptic concentration of NA. By contrast, P2Y6Rs of glutamatergic nerve terminals down-regulate the uptake of glutamate (vesicular glutamate transporter 1; VGLUT1). All these effects lead to a modulation of neuronal excitability and synaptic functions through intracellular signaling pathways to maintain neuronal circuit homeostasis. Although we indicated that P2Y6Rs are located at the terminals of noradrenergic and glutamatergic neurons, it is quite possible, that the localization of these receptors is exclusively microglial. Eventually, P2Y6Rs upregulate lysosomes in key regions of the CNS and in consequence enhance microglial degradation

P2Y6Rs play an integral role in the complex process of synaptic pruning (Fig. 1A). The first 3 weeks of mouse life mark the peak period of synaptic remodeling and pruning, characterized by significant upregulation of microglial activity and P2Y6R expression. P2Y6Rs are thought to be involved in multiple aspects of somatosensory cortical maturation, including dendritic reorganization of layer 4 neurons of the cerebral cortex [54].

By postnatal day 30 (P30), the presynaptic marker vesicular transporter 1 (VGLUT1) was markedly reduced in the brain tissue of P2Y6R knockout mice, resulting in a significant decrease in the uptake of the excitatory transmitter glutamate in the somatosensory cortex, hippocampal CA3 area, and dentate gyrus. In addition, P2Y6R knockout adult mice exhibit significant deficits in short-term and long-term spatial and recognition memory [28]. In PS1/APP mice, modelling Alzheimer’s disease, activation of P2Y6Rs could restore synaptic plasticity, thereby reversing hippocampus-dependent contextual memory deficits [55]. These compelling findings highlight that by clearing unnecessary synapses and regulating neuronal connections, P2Y6Rs can influence the shaping of neural circuits, promoting the formation of stable and efficient synaptic networks.

### P2Y6 Receptor Regulates Neurotransmitter Turnover

The roles of P2Y6Rs in the nervous system are multifaceted. In addition to mediating synaptic pruning of neural circuits, emerging evidence suggests that P2Y6Rs also play a key role in regulating neurotransmitter release, thereby affecting communication between neurons and neuroplasticity (Fig. 1B). Purinergic signaling has been shown to inhibit NMDAR-induced norepinephrine release in the cerebral cortex, potentially modulating NMDA-driven neuroplasticity and providing neuroprotection to noradrenergic circuits [56]. Activation of different purinergic receptors, such as purinergic P2Y1, P2Y6, and adenosine A1, and A2Rs, can suppress NMDA-induced noradrenaline release through different mechanisms. In particular, P2Y6Rs may play a key role in regulating norepinephrine transmission by facilitating communication between neurons and perisynaptic glia, thereby indirectly inhibiting noradrenergic transmission, as a consequence of increased noradrenaline uptake into astrocytic cells [13, 57]. Noradrenaline affects neuronal membrane potential and ion channel activity by regulating supposedly via β-adrenoceptors and related intracellular signaling pathways such as cyclic adenosine monophosphate (cAMP)-regulated protein kinase A (PKA) and mitogen-activated protein kinase (MAPK) pathways [58]. These events play an important role in the orchestration of cognitive processes such as learning and memory, as well as the underlying neuroplasticity processes [59]. Therefore, P2Y6R-mediated control of neurotransmitter release interferes with signal transduction and neuronal communication, affects synaptic excitability and plasticity, maintains the stability of the nervous system, and contributes to the remodeling and optimization of neuronal circuits.

## Microglial P2Y6 Receptors Control Synaptic Dysfunction: The Role of P2Y6 Receptors in Neurodegenerative Diseases

Because of the importance of axonal and dendritic pruning during development and adult life, these remodeling events require precise control, which is achieved by several different molecular mechanisms. Disruption of these mechanisms leads to abnormal pruning and ultimately brain dysfunction [60]. Any abnormality related to P2Y6R function can lead to neurodevelopmental disorders [61]. Activation of P2Y6Rs helps to optimize brain structure and cognitive performance, whereas inappropriate regulation of P2Y6R signaling may disrupt the delicate balance between synapse formation and clearance, leading to developmental disorders or neurodegenerative diseases. Upregulation of P2Y6Rs can lead to excessive pruning, causing neural circuits to lose necessary connections, and impairing learning and memory processes that rely on these pathways. Conversely, insufficient pruning due to a reduction in P2Y6Rs can lead to excessive or inefficient synapses in the brain, disrupting the optimization of neural circuits.

In the following, we will discuss some primary (Alzheimer’s disease, Parkinson’s disease, neuropathic pain) and secondary (epilepsy, ischemic-, mechanistic-, or irradiation-induced) forms of neurodegeneration and the involvement of P2Y6R in these diseases or afflictions.

### P2Y6 Receptors in Alzheimer’s Disease

As the brain ages, microglial activity increases, which can lead to excessive clearance and phagocytosis of synapses. This excessive phagocytosis of synapses reduces the connections between neurons, thereby affecting brain function and specifically cognition, and P2Y6Rs play a key role in this process [62]. Studies have shown that the expression of P2Y6Rs is increased in aged mice, which show reduced synaptic density in the cortex and hippocampus, leading to memory impairment and brain atrophy, which is attributed to increased internalization of synaptic components by microglia [63]. In contrast, P2Y6R knockout mice did not show synaptic loss, neuroinflammation, age-related synaptic decline, memory deficits, or cognitive impairment. Alzheimer’s disease (AD) is an age-related neurodegenerative disease characterized by significant neuronal degeneration and damage, particularly in the hippocampus and cerebral cortex, key brain regions involved in memory and cognition [64, 65]. In this disease, the neurotoxic β-amyloid (Aβ) peptide aggregates in the extracellular space (neurofibrillary tangles), and hyperphosphorylated tau-protein is accumulating intracellularly in neurons [66]. Recent evidence suggests that inhibition of the P2Y6R may offer a promising therapeutic avenue for patients with AD. In an Aβ-treated mouse model, blocking P2Y6Rs with the antagonist MRS2578 significantly reduced phagocytosis in microglia-neuron co-cultures [25]. Furthermore, studies in models of acute Aβ-induced dementia and chronic tau pathology in P2Y6R knockout mice showed attenuated neuronal loss and preserved memory function compared to wild-type mice [62].

In contrast, in 5XFAD mice (a genetic model of AD), specifically in amyloid plaque-associated microglia, the P2Y6R response to UDP was significantly attenuated, resulting in reduced microglial phagocytosis [67]. This damage may impair the ability of microglia to clear amyloid deposits and cellular debris. These apparently contradictory findings highlight the complexity and dynamics of microglial function during Alzheimer’s disease pathogenesis. The role of the P2Y6R in Alzheimer’s disease appears to be multifaceted and may play a protective or pathogenetic role depending on the stage of the disease. This complexity suggests that precise modulation of P2Y6R activity (rather than complete inhibition of activation) may be a promising therapeutic strategy to address brain aging and AD-related pathologies.

### P2Y6 Receptors in Parkinson’s Disease and Neuroinflammatory Damage

Hallmark pathological changes in Parkinson’s disease (PD) are intra-neuronal and intra-axonal α-synuclein-positive inclusions (Lewy bodies, Lewy neurites) and loss of dopaminergic neurons in the substantia nigra pars compacta and their projection areas in the striatum [68, 69].

The critical regulatory role of P2Y6Rs in microglia phagocytosis is widely recognized. However, P2Y6R-mediated signaling in microglia also has a major impact on cytokine and chemokine production. Recent studies have demonstrated that P2Y6R activation markedly amplifies the pro-inflammatory reaction initiated by Toll-like receptors (TLRs) [70] resulting in the transcriptional activation of extracellular signal–regulated kinases (ERK), as well as those of nuclear factor of activated T-cells (NFAT), immature reticulocyte factor (IRF), and NF-κB, thereby influencing the secretion of a spectrum of cytokines and chemokines, such as IL-1β, IL-6, IL-8, TNF-α, and monocyte chemoattractant protein-1 (MCP-1) [71, 72]. This cascading response leads to neuroinflammation, which ultimately results in neuronal loss and neurodegeneration. Specifically, neuroinflammation impairs neuronal and glial cell function, disrupts synaptic transmission, and interferes with normal neural circuitry and information processing. Inhibition of microglia activity and modulation of P2Y6R expression provide excellent ways to alleviate neuroinflammation and inflammation-induced neurological disorders.

Studies support the hypothesis that the loss of nigrostriatal dopaminergic neurons in PD may be influenced by peripheral lipopolysaccharide (LPS), which significantly increases the expression of inflammatory cytokines and P2Y6Rs. Microglial P2Y6Rs were shown to regulate neuroinflammation triggered by LPS-induced UDP secretion, promote autocrine recycling, and accelerate neuroinflammatory pathways via ERK1/2. Notably, P2Y6R knockout mice did not show LPS-induced neuronal loss [73–75]. Moreover, in the 6-hydroxydopamine (6-OHDA)-treated rat model of PD (6-OHDA injection into the right medial forebrain bundle leads to the unilateral degeneration of dopaminergic neurons), selective P2Y6R antagonism by MRS2578 prevented dopaminergic neurodegeneration and microglial activation in the substantia nigra [76]. Furthermore, in another PD model utilizing rotenone, an inhibitor of mitochondrial complex I, both microglial activation and dopaminergic neuron loss were observed. Notably, rotenone-induced neurotoxicity is mediated, at least partially, through microglial phagocytosis of otherwise viable neurons. P2Y6Rs appear to play a pivotal role in this process, and their antagonist, MRS2578, demonstrates neuroprotective effects by inhibiting microglial phagocytosis [77].

Unfortunately, the authors limited their study to the investigation of cellular changes and did not extend it to the measurement of the motor symptoms of PD. Similarly, 1-methyl-4-phenylpyridinium (MPP^+^), which selectively destructs dopaminergic neurons of the brain without affecting noradrenergic neurons alike 6-OHDA, increased the level of P2Y6Rs in the neuroblastoma SH-SY5Y cell line [78].

Thus, microglial activation and elevated levels of pro-inflammatory cytokines characterize the PD brain, and the blockade of P2Y6Rs has a possible therapeutic value as a treatment strategy for PD patients.

### P2Y6 Receptors and Neuropathic Pain

Neuropathic pain (NP) has the cardinal symptoms of spontaneous, continuous, or paroxysmal pain, hypersensitivity to painful stimuli (lower threshold), and allodynia. Nervous system disease or damage, such as trauma and injury of the peripheral or central nervous system, viral infection, metabolic dysregulation, and ischemia, is the general cause of neuropathic pain [79]. A prominent signaling pathway in the development of neuropathic pain involves ATP acting on microglial purinergic receptors.

Studies utilizing the chronic constriction injury (CCI) rat model of NP demonstrated that the P2Y6 receptor agonist UDP exacerbates it, suggesting a crucial role for P2Y6R in NP modulation [80, 81]. Peripheral nerve injury in rats, as another model of neuropathic pain, induced a dramatic increase of P2Y6R-mRNA in spinal microglia [80, 82]. Accordingly, the P2Y6R antagonist MRS2578 attenuated pain hypersensitivity. Intrathecal injection of the P2Y6R agonist UDP caused mechanical allodynia and thermal hyperalgesia in naïve rats and enhanced these symptoms of neuropathic pain in rats pre-treated with CCI of the left sciatic nerve [82]. Spinal nerve ligation also caused tactile allodynia in rats and increased the expression of P2Y6Rs in spinal microglia [83]. Intrathecal administration of MRS2578 again reduced the increased receptor expression and the prominent tactile allodynia. These findings indicate that alterations in neuron-microglia interactions are causal factors in the development of P2Y6R-mediated neuropathic pain. This hypothesis is further supported by the observation that the broad-spectrum anti-inflammatory drug minocycline has a beneficial effect on tactile allodynia and mitigates the nerve injury-induced upregulation of Iba1-positive microglia in a rat model of NP [83, 84].

### P2Y6 Receptors in Epilepsy

Under normal circumstances, there is a balance between excitatory and inhibitory neurons in the brain. However, in patients with epilepsy, this balance is disturbed, leading to the overactivation of excitatory neurons, which trigger epileptic seizures [85]. Neuroinflammation is increasingly considered a pathogenetic factor in epilepsy [86]. The hippocampus-prefrontal cortex circuitry is involved in regulating higher-order functions such as memory and cognition and plays a critical role in epilepsy [87]. Recent studies have increasingly demonstrated that purinergic signaling through P2 receptors plays a pivotal role in the exacerbation of pathological states associated with hyperexcitability and epilepsy. It is noteworthy that an increase in P2Y6R expression has been observed in the hippocampus and cortex during kainate- and pilocarpine-induced status epilepticus [88, 89] where P2Y6R activation regulates lysosomal expression and promotes neuronal phagocytosis, indirectly affecting the excitability of hippocampal and prefrontal cortex neurons [29].

Interestingly, UTP has potent anticonvulsant and neuroprotective properties during prolonged epileptic seizures [90]. However, the upregulation of P2Y6Rs in epilepsy patients leads to reduced levels of UTP, which attenuates neuroprotection by competing with the endogenous agonist UDP. In addition, increased spontaneous calcium activity in microglia is a common feature of epilepsy and is primarily controlled by P2Y6R signaling [29]. Notably, P2Y6R knockout mice performed better on cognitive tasks during early-onset epilepsy, possibly due to a significant reduction in neuroinflammation and cell loss caused by P2Y6R activation during this period. Interventions targeting P2Y6Rs may also play a role in the treatment of epilepsy, improving cognitive function by reducing neuroinflammation and cell loss.

### The Role of P2Y6 Receptors in the Repair of Brain Injury

In the case of acute brain injury, microglial phagocytosis may be beneficial in promoting axonal regeneration and restoring the microenvironment, thereby aiding recovery from brain injury by rebuilding neuronal networks in the brain [91]. Specifically, P2Y6R-mediated phagocytosis by microglia plays a key role in this process, helping to clear damaged cells and debris and maintain neuronal connections and circuits, thereby promoting neuroplasticity after brain injury.

Studies have shown that P2Y6R expression is significantly upregulated in microglia following transient middle cerebral artery occlusion (ischemic stroke model [92]). In particular, selective inhibition of P2Y6Rs using the inhibitor MRS2578 has been shown to exacerbate brain atrophy and edema volume after ischemic stroke by inhibiting microglial phagocytic activity. After stroke, there is a delayed neuronal loss in brain areas surrounding the infarct, which appears to be mediated at least partly by microglial phagocytosis of stressed neurons [75]. It is generally believed that this phagocytosis is initiated by UDP released from injured neurons activating P2Y6Rs at microglia. In confirmation of this idea, when wild-type mice were subjected to transient focal ischemia, there was pronounced neuronal loss in the peri-infarct area, whereas P2Y6R knockout mice did not exhibit any comparable damage. Finally, in a model of intracerebral hemorrhage in mice an inflammatory variant of apoptosis, termed pyroptosis was observed in microglia [93].

In contrast, activation of P2Y6Rs was found to be neuroprotective in radiation-induced brain injury. Specifically, activation of P2Y6Rs protects neurons and promotes remyelination by enhancing microglial clearance of necrotic cells and debris. Conversely, blocking P2Y6Rs inhibits the radiation-induced phagocytic activity of microglia, leading to increased neuronal apoptosis and impaired remyelination [94]. Taken together, the P2Y6R emerges as a promising therapeutic target for the development of strategies aimed at enhancing the brain’s intrinsic repair mechanisms and facilitating functional recovery in the aftermath of acute brain insults.

## Conclusions

Activation of P2Y6R has been demonstrated to exert a significant regulatory influence on the function of microglia, thereby exerting a profound impact on neural circuits. Specifically, P2Y6R functions through two key mechanisms: (1) stimulating synaptic phagocytosis of microglia, thereby directly participating in the remodelling of neural circuits; (2) regulating the levels of neurotransmitters and inflammatory mediators, which indirectly affect the activity of neurons and microglia. These mechanisms collectively regulate the homeostasis of neural circuits.

Recent studies have revealed the crucial role of P2Y6R in various stages of the nervous system development. During early postnatal brain development, it plays a pivotal role in the initial formation and consolidation of neural circuits. In adulthood, it is essential for maintaining neural plasticity. Furthermore, in the context of aging and neurodegenerative diseases, it interacts with neural circuits. Disruption of homeostasis is closely associated with these processes. These findings provide crucial insights into the regulatory mechanisms of neural circuit functions during learning and memory. In light of the pivotal function of P2Y6R in the modulation of neural circuits, future research should prioritize a comprehensive investigation into the molecular mechanisms through which P2Y6R influences microglia-mediated neural circuit remodeling. Additionally, it is imperative to elucidate the spatiotemporal expression pattern of P2Y6R across different developmental stages and brain regions. In conclusion, research into the role of P2Y6R in various neurological diseases provides a basis for future treatment options.

## Data Availability

No datasets were generated or analysed during the current study.
